# Sensitivity Analysis of Factors Influencing Blast-like Loading on Reinforced Concrete Slabs Based on Grey Correlation Degree

**DOI:** 10.3390/ma16165678

**Published:** 2023-08-18

**Authors:** Zhixiang Xiong, Wei Wang, Yangyong Wu, Wei Liu

**Affiliations:** 1 Key Laboratory of Advanced Ship Materials and Mechanics, Harbin Engineering University, Harbin 150001, China; xiongzx@hrbeu.edu.cn; 2 Department of Engineering Mechanics, College of Aerospace and Civil Engineering, Harbin Engineering University, Harbin 150001, China; 3 Key Laboratory of Impact and Safety Engineering, Ministry of Education, Ningbo University, Ningbo 315211, China; 4 School of Safety Science and Engineering, Anhui University of Science and Technology, Huainan 232001, China; 5 Institute of Engineering Safety and Disaster Prevention, Hohai University, Nanjing 210098, China

**Keywords:** blast simulator, impact loading, reinforced concrete slabs, grey correlation, sensitivity analysis

## Abstract

Blast simulators are capable of applying blast-like loading to components in a safe and controlled laboratory environment, overcoming the inherent shortcomings of blast testing in terms of data acquisition, test cycle time, and cost. In this paper, reasonable assumptions and refinements are made to the components and shape of the impact module, a key component of the blast simulator, to achieve diversity in simulated blast loading. By designing four rubber shapes, the importance of ellipsoid rubber as an elastic cushion for simulating blast loading was determined. In order to assess the effectiveness of this optimization, numerical calculations based on a calibrated finite element model were performed around four factors: flat rubber thickness, ellipsoid rubber thickness, impact velocity, and impact modulus mass. Additionally, a grey correlation sensitivity analysis was carried out to evaluate the effect of these factors on the impact loading on the reinforced concrete (RC) slab. The results indicate that peak pressure and impulse had opposite sensitivities to velocity and mass. Changes in ellipsoid rubber thickness had a more positive effect on the impact loading than flat rubber thickness. An in-depth study of the role of these influencing factors is important for the design and improvement of impact modules.

## 1. Introduction

In recent years, ongoing terrorist attacks have demonstrated the vulnerability of critical infrastructures such as energy, health, communications, government, and transport to the threat of traditional explosives and improvised explosives. As a result of factors such as the weakening of the United States globalized counter-terrorism strategy, the continuing global new crown epidemic, and the global economic downturn, the development of global terrorism has become more complex, and the global counter-terrorism situation has become even more critical. In order to safeguard the security and stability of society, governments are faced with serious challenges, and there is an urgent need to carry out research on the destructive effects of structural components in explosions, as well as reinforcement and modification of existing structures. Therefore, it is of great significance to develop an efficient, convenient, safe, and reliable means of structural blast resistance testing.

The research and development and large-scale application of new explosion-proof structures cannot be separated from the study of explosive loads. Explosive effect research is generally based on the field explosion test, combined with numerical simulation and theoretical analysis, so the field explosion test in the explosion effect research occupies an important position. As a traditional means of explosive loading research, the chemical explosion test can directly apply the real explosive load on the structure or component, a true reflection of its resistance to the explosion [1,2,3,4,5,6,7]. Schenker A [6] carried out explosion tests on concrete slabs in their original dimensions and obtained test data on the dynamic response of concrete members and verified them using numerical calculations. However, there are some problems in the field chemical explosion test. Firstly, it requires a lot of test funds and time. Secondly, it is difficult to visualize and take high-speed photographs of the structural damage process due to the fireball generated by the explosive explosion. More importantly, due to the strong non-linearity of the explosion dynamics and the complexity of the kinetic properties of the target plate material, it is difficult to obtain repeatable, stable, and reliable test data from this method.

In the face of the numerous shortcomings that cannot be avoided in traditional explosion tests, non-explosive methods that are safe and controllable and can be studied in the laboratory have emerged. Kumar et al [8] investigated the drop impact resistance and energy absorption capacity of prestressed concrete slabs through experimental analysis and numerical calculations. In addition, to investigate the effect of initial stress on the impact response, Kumar [9] also compared the calculated results of prestressed concrete slabs directly with reinforced concrete slabs of equivalent thickness. However, existing studies have shown that, due to the free-fall loading method, the impact velocity of the drop hammer is generally low due to the height limitation of the equipment, which cannot effectively simulate the explosion load waveform. On the other hand, due to the limitation of the size of the hammer, the drop hammer can generally only locally load materials and small-sized components. In addition, the secondary impact caused by the rebound of the falling hammer seriously affects the realism of the waveform [10,11]. The shock tube can generate loads with a long enough loading time, which is suitable for simulating the explosion loads in the middle and far regions, but it is difficult to simulate the explosion loads in the near region, and only a few laboratories in the world have large shock tubes to meet the conditions. Mr. Li [12] used experimental and numerical methods to investigate the dynamic loading characteristics of steel-tube concrete columns subjected to proximity blast loading, and evaluated and discussed the effects of charge setting and axial loading on the structural response and damage. In addition, the loading area of the surge tube is limited by the cross-sectional diameter of the shock tube [13,14]. Similar to the shock tube, the specimen size for light air gun tests is also affected by the diameter [15,16]. Chen et al. [15] dynamically loaded small composite beam specimens by firing compressible foam projectiles at velocities of 30 to 60 m/s using a gas gun to investigate the dynamic damage characteristics of beam members under blast loading. The University of California, San Diego, has developed a blast load simulation device called the UCSD Blast Simulator [17]. Since its introduction in 2005, the UCSD Blast Simulator has been used to study the blast resistance of a variety of masonry walls, high performance blast walls, concrete columns, steel columns, and composite structures. Freidenberg [18] demonstrated the ability of a blast simulator to generate blast loads on structures by comparing blast simulation tests, numerical simulations, and in situ blast tests. The European Laboratory for Structural Assessment (ELSA) has constructed a novel test facility called the Electronic Blast Simulator (e-BLAST), which utilizes servo-controlled drive technology to simulate the exposure of structures to air shock wave loading without the use of explosives. Recently, based on the blast simulator, Xiong et al. [19] conducted two sets of impact tests on reinforced concrete slabs to investigate the effects of the shape of the elastic cushion and the impact velocity on the impact loading characteristics, respectively. The results show that the newly developed blast simulator, which is capable of applying explosive-like loads to reinforced concrete slabs, achieves the goal of mechanical impact as an equivalent alternative to chemical explosion tests.

In order to achieve diverse simulation of blast loading using a blast simulator, this paper improves the impact module and performs numerical calculations around four factors: impact velocity, impact module mass, flat rubber thickness, and ellipsoid rubber thickness. To further investigate the sensitivity of impact loading to different factors, this paper employs grey correlation analysis to evaluate the sensitivity of these factors to the impact loading characteristics. This work is meaningful in that several optimal designs have been achieved through numerical calculations, which resulted in significant savings in project cost and testing time.

## 2. Grey Correlation Analysis Method

The grey correlation analysis method is an important sensitivity analysis method. The basic idea of this method is based on the grey theory; the variables in the system are regarded as factors with different importance and degree of correlation. The data are transformed into a grey correlation series by performing grey transformation and standardization of the data. The degree of correlation between the different series is then assessed by calculating the correlation coefficient or correlation degree index. In grey correlation analysis, the correlation coefficient is usually used to indicate the degree of association between variables. The value of correlation coefficient ranges from 0 to 1. the closer to 1, the higher degree of correlation; the closer to 0, the lower degree of correlation. The grey correlation analysis method can be used to analyze and evaluate the influencing factors of impact load peak pressure and impulse. The specific calculation steps of the method are as follows:

(1) Determination of the sequence matrix

The factors affecting the peak load pressure (impact velocity *V*, impact modulus mass *M*, flat layer rubber thickness *h*_1_, and ellipsoid rubber thickness *h*_2_) are selected as the influence factor subsequence *X*, *X = (X X*_12_*...X)_i_^T^*, the corresponding peak load pressure as the parent sequence *Y*, *Y = (Y Y*_12_*...Y)_i_^T^*, and the load impulse as the parent sequence *Z*, *Z = (Z Z*_12_*...Z)_i_^T^*. Each factor of series *X*, series *Y,* and series *Z* has *j* values and the matrix form

(1) $$X=\left(\begin{array}{c} X_{1}\\ X_{2}\\ \vdots \\ X_{i} \end{array}\right)=\left(\begin{array}{cccc} x_{11} & x_{11} & \cdots & x_{11}\\ x_{21} & x_{22} & \cdots & x_{2j}\\ \vdots & \vdots & \ddots & \vdots \\ x_{i1} & x_{i2} & \cdots & x_{ij} \end{array}\right),$$

(2) $$Y=\left(\begin{array}{c} Y_{1}\\ Y_{2}\\ \vdots \\ Y_{i} \end{array}\right)=\left(\begin{array}{cccc} y_{11} & y_{11} & \cdots & y_{11}\\ y_{21} & y_{22} & \cdots & y_{2j}\\ \vdots & \vdots & \ddots & \vdots \\ y_{i1} & y_{i2} & \cdots & y_{ij} \end{array}\right),$$

(3) $$Z=\left(\begin{array}{c} Z_{1}\\ Z_{2}\\ \vdots \\ Z_{i} \end{array}\right)=\left(\begin{array}{cccc} z_{11} & z_{11} & \cdots & z_{11}\\ z_{21} & z_{22} & \cdots & z_{2j}\\ \vdots & \vdots & \ddots & \vdots \\ z_{i1} & z_{i2} & \cdots & z_{ij} \end{array}\right).$$

(2) Matrix dimensionless

The dimensionless methods of matrix are initialization, homogenization, interval relativization, and normalization. In this paper, interval relativization is used to process the raw data, mapping the data to the range of 0 to 1 and eliminating the dimension differences:

(4) $$X_{i}^{\prime }=(x_{i1}^{\prime }x_{i2}^{\prime }\cdots x_{ij}^{\prime }).$$

Among them

(5) $$x_{ij}^{\prime }=\frac{x_{ij}-\min x_{ij}}{\max x_{ij}-\min x_{ij}}.$$

Similarly, the reference matrices *Y* and *Z* can be made dimensionless in the same way.

(3) Differential sequence matrix

The dimensionless subsequence is processed using the parent sequence in the following manner to obtain the difference sequence matrix Δ. The maximum and minimum values are then extracted from it:

(6) $$\Delta_{ij}=\left|y_{ij}^{\prime }-x_{ij}^{\prime }\right|,$$

(7) $$\{\begin{array}{c} \Delta_{max}=max(\Delta_{ij})\\ \Delta_{min}=min(\Delta_{ij}) \end{array}.$$

(4) Grey correlation coefficient matrix

The factors of the grey correlation coefficient matrix *L* were calculated using the following equation:

(8) $$l_{ij}=\frac{\Delta_{min}+\varphi \Delta_{max}}{\Delta_{ij}+\varphi \Delta_{max}}$$

where *φ* is the resolution factor, *φ* ∈ [0, 1], and *φ* = 0.5 is taken in general.

(5) Solve for the grey correlation *G*

Solving the average value of the grey correlation coefficients in each row of the grey correlation coefficient matrix, and taking this average value as the correlation degree of the influencing factors of the corresponding row, can solve the disadvantage of the number of correlation coefficients which is large and scattered; the correlation degree calculation formula is

(9) $$g_{i}=\frac{1}{m}\sum_{j=1}^{m}\gamma_{ij}.$$

The correlation takes the value range of [0, 1] and the order of correlation value reflects the sensitivity of the influencing factors; the larger the value of correlation, the stronger the correlation between the comparing factors and the reference factors.

## 3. Modelling of Blast Loading

### 3.1. The Basics of Blast Loading

**Figure 1:**
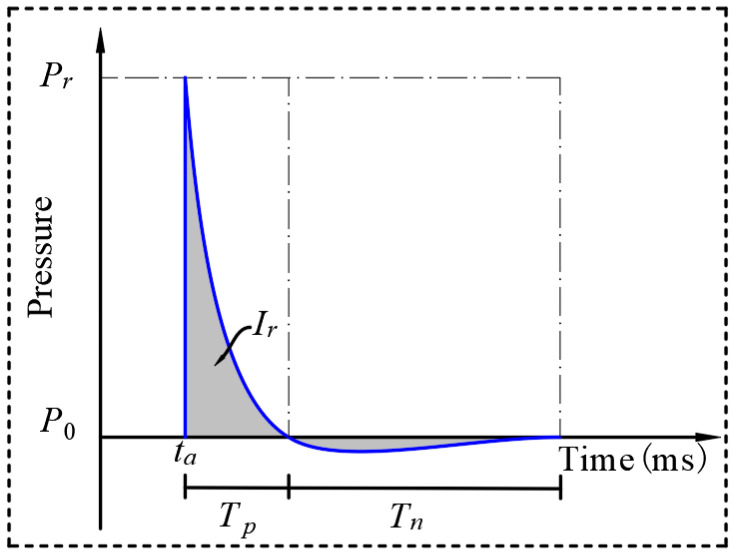
Typical blast reflection pressure–time-course curve.

The detonation of spherical TNT produces a shock wave that forms a reflected wave when it reaches the surface of the structure. A typical reflected pressure profile at a point on the structure is shown in Figure 1. Its shape shows a sharp increase in pressure followed by an exponential decay. The explosive detonation shock wave wavefront to reach the target time is known as the arrival time $T_{0}$. At this time, the shock wave pressure takes a very short period of time to the maximum pressure, and then quickly drops to atmospheric pressure $P_{0}$; $T_{a}$ is the positive phase duration. $P_{r}$ is the peak of the reflected overpressure, i.e., the pressure–time curve enclosing the area impulse $I_{r}$. The pressure and impulse can be calculated in a number of ways, including using WU, C.’s formula [20] and consulting the TM5-1300 manual [21].

### 3.2. Blast Similarity Law

The Law of Explosive Similarity is a theory used in the study of blast wave propagation to describe the similarity between different explosive events under certain conditions. The principle states that if two explosive events have the same proportionality in certain key parameters (e.g., mass, energy, distance, etc.) the blast effects between them will be similar. This method allows tests to be carried out under relatively safe conditions, avoiding the risks that may be associated with the direct use of large quantities of explosives for testing.

(10) $$Z=\frac{R}{\sqrt[{3}]{W}},$$

(11) $$W=\frac{Q_{EXP}}{Q_{TNT}}W_{EXP}$$

where *Z* is the proportional distance, *R* is the distance between the TNT charge and the target plate, *Q_EXP_* is the energy of the explosives, *Q_TNT_* is the energy of the TNT, *W* is the mass of the TNT charge, and *W_EXP_* is the mass of the charge.

### 3.3. Blast Load Parameters

The basic parameters of explosive loading are peak overpressure, impulse, duration, and the decay relationship (curve shape) of overpressure–time. In the finite element method (FEM), a variety of methods can be used to simulate the explosive load, including quasi-static analysis, explosive loading model, and fluid–solid coupling analysis. The CONWEP model is a common explosive loading model, which calculates the explosion waveform and propagation characteristics by considering the characteristic parameters of the blast loading (e.g., charge, blast distance, etc.), and then applies them to a specific area or surface in the finite element model [22]. For numerical calculations, only the target plate needs to be modelled and no air domain mesh is required. A *SET_SEGMENT pressure loading surface is created on the blast-facing side of the target plate to define the shock wave pressure loading region. Add * LOAD_BLAST and * LOAD_SEGMENT to the K file to define the weight of the spherical TNT W, the distance from the structure of the blast face R; add the keywords * DATABASE_ELOUT and * DATABASE_HISTORY_SOLID to define the pressure output. The model only needs to define the mass of the TNT, the coordinates of the explosion center, and the time of detonation.

**Figure 2:**
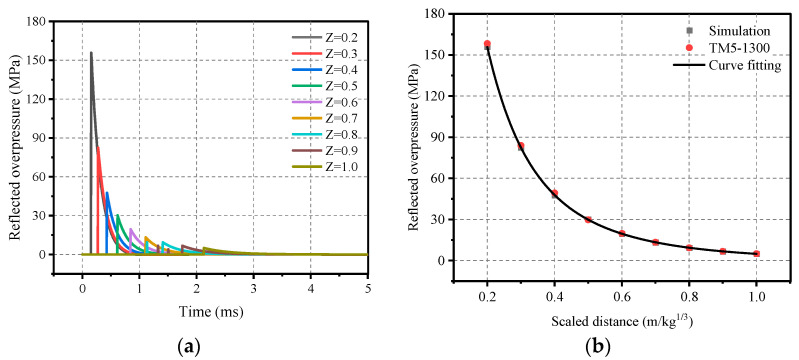
Spherical TNT airburst shock wave: (**a**) Reflected overpressure–time-course curve; (**b**) Positive reflected overpressure peaks.

**Figure 3:**
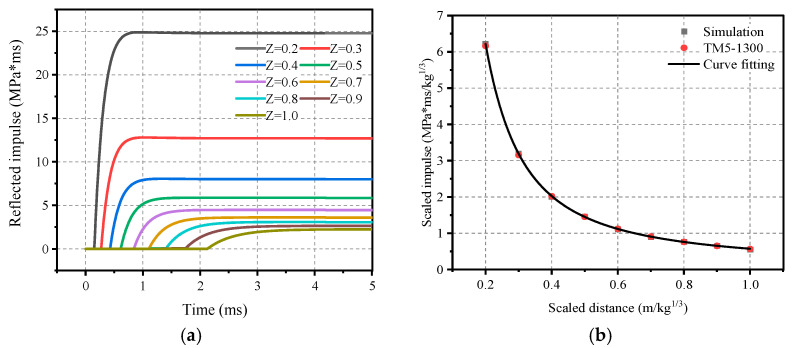
Spherical TNT airburst shock wave: (**a**) Positively reflected impulse time-course curve; (**b**) Proportional impulse peaks.

In order to facilitate the calculation, in accordance with the law of similarity of the explosion, the design of the following conditions is required: the charge mass of 64 kg and the explosion distance range of 0.8 m to 4 m, that is, the proportion of the distance between 0.2 and 1.0. Figure 2b and Figure 3b depict the results of the calculations with the TM5-1300 manual [21]. The comparison of the calculated results is more credible. Therefore, the peak overpressure and impulse were fitted to facilitate a quick calculation of the required explosion conditions, and the formulas are shown in respectively (12) and (13).

(12) $$\Delta P_{r}=3.413-14.085/Z+17.713/Z^{2}-2.218/Z^{3}+0.092/Z^{4},\;\;\;\mathrm{MPa}$$

(13) $$I_{r}=W^{1/3}(0.114/Z+0.023/Z^{2}-0.006/Z^{3}-0.0006/Z^{4}),\;\;\mathrm{MPa}\cdot \mathrm{ms}$$

Scope of application: spherical TNT airbursts; proportional distance 0.2 ≤ Z ≤ 1.0.

## 4. Finite Element Modelling

### 4.1. Calibrated Model

**Figure 4:**
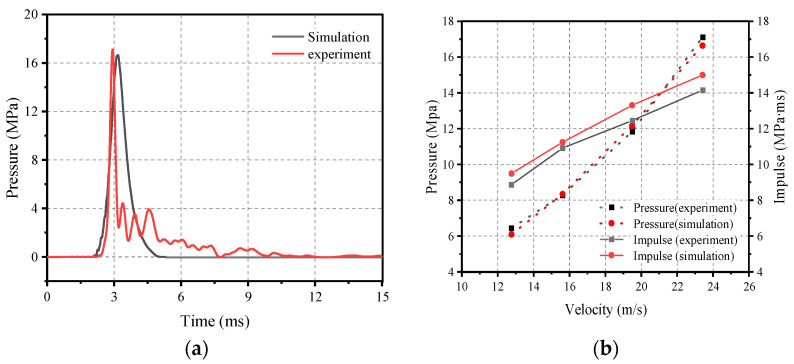
Comparison of numerical and experimental results: (**a**) Pressure curve (v = 23.41 m/s); (**b**) Peak pressure and impulse [20].

A new impact-based facility has been developed that vertically launches multi-mass impact modules to load large-size members at precisely high velocities (VMLH). Xiong Zhixiang et al. [19] used the VMLH Blast Simulator to conduct two sets of impact tests: one set of tests selected a range of flat rubber thicknesses from 20 to 100 mm and impact velocities of approximately 15 m/s; the other set used conical rubbers arranged in a 5 × 5 arrangement, with impact velocities set from 10 to 25 m/s. The results show that prismatic conical rubber is more suitable than flat rubber as an impact pad for simulating blast loads. Based on this experimental conclusion, Xiong et al. developed a numerical model for the impact conditions of prismatic conical rubber as an impact pad, and calibrated the numerical model by comparing the results of numerical calculations and experimental tests. Figure 4a,b shows the pressure–time curve at a velocity of 23.41 m/s and the peak pressure and impulse of the second set of tests, respectively.

Based on the experimentally validated finite element model, the position of the rubber was adjusted to attach it to the underside of the steel plate, and the driver was used to preload the impact module with an adjustable velocity, which in turn achieved the goal of generating a blast-like loading on the specimen through the impact module. The process of impact is shown in Table 1, where the impact module consists of two parts: a counterweight steel plate and an impact cushion. Use a counterweight steel plate as the mass of the entire impact module body for impact loading to provide enough adjustable kinetic energy, so as to control the peak impact load. Fix an elastic pad layer in the front of the impact module, directly in contact with the target; this prevents damage to the metal materials in the module. This also enables the impact of the module and the target of the flexible contact to prolong the loading time; more importantly, its viscoelastic material properties determine the waveform of the load transmitted by the impact.

### 4.2. Improved Model

**Figure 5:**
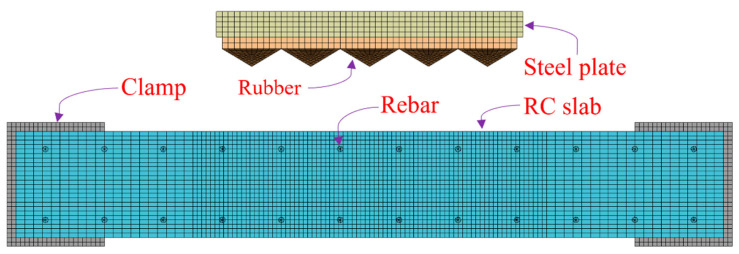
Reinforced concrete slab impact finite element model.

**Figure 6:**
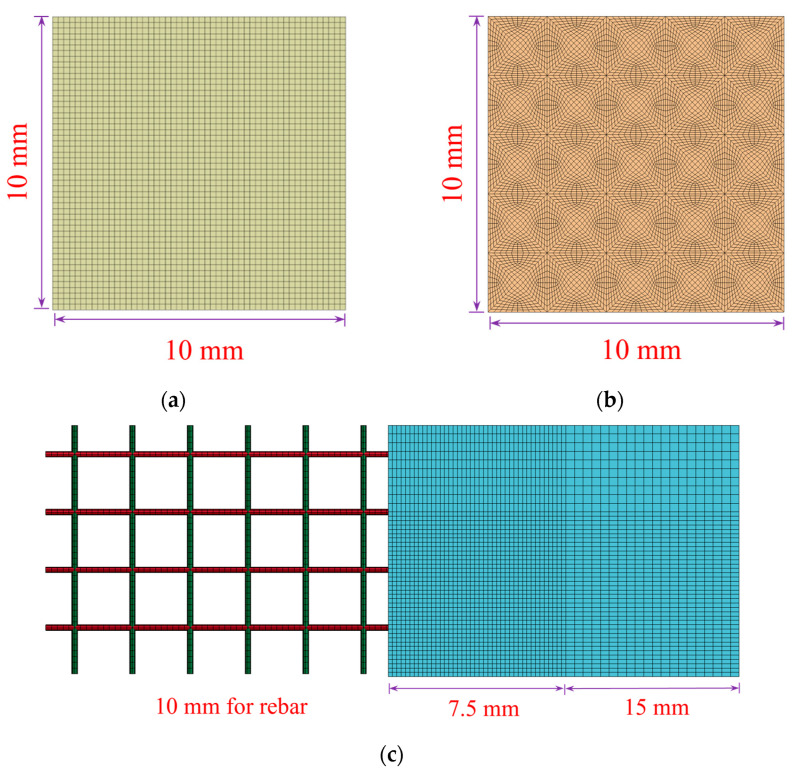
Details of meshing: (**a**) Steel plate; (**b**) Rubber; (**c**) Reinforced concrete plate.

The finite element model is shown in Figure 5, where the rubber is bonded to the bottom of the steel plate and collides with the RC plate after accelerating to a specified velocity. The model includes a reinforced concrete plate, rubber, counterweight steel plate, and fixture. An eight-node solid hexahedral cell is used for meshing, and reference is made to the mesh convergence analysis performed by the previous subject group [19]; a concrete mesh size of 7.5 mm was used, doubled outside the range of ±600 mm from the center of the slab to reduce the computation time. The cell size of the impact module was 10 mm in both the edge length and thickness directions. The upper part of the rubber was prismatic and tangential to obtain a mesh size of approximately 10 mm. The meshing details are shown in Figure 6.

### 4.3. Material Modelling and Parameters

To obtain correct numerical results, reasonably correct material models and material parameters must be used in modelling.

#### 4.3.1. Concrete

The concrete material was used in the concrete continuous surface cap model *MAT_CSCM_CONCRETE developed by the Federal Highway Administration (FHA) organization to simulate the dynamic performance of reinforced concrete roadside protection structures under the influence of vehicle collisions [23]. The continuous face cap model takes into account material hardening, damage and rate dependence, and is currently widely used in the field of low-speed impacts on reinforced concrete structures and can be used in numerical simulations with relatively simple input modes. The concrete material in the present study is shown in Table 2.

#### 4.3.2. Steel

Reinforcement bars are reinforced using the plasticity-following reinforcement model *MAT_PLASTIC_KINEMATIC, based on the factory test report of the material provided by the rebar manufacturer; the material parameters are as follows Table 2. Considering the influence of strain rate effect on the intrinsic relationship of the materials, the expression of dynamic yield strength of steel is shown in Equation (14).

(14) $$\sigma_{y}=\left[1+{\left(\frac{\overset{\cdot }{\varepsilon }}{C}\right)}^{1/P}\right]\left(\sigma_{0}+\beta E_{P}\varepsilon_{P}{}^{eff}\right)$$

where $\sigma_{y}$ is the dynamic yield strength of steel; ε is the strain rate; *C*, *P* are the strain rate parameters; σ_0_ is the initial yield strength of steel; *β* is the hardening parameter; $E_{P}$ is the hardening modulus; and $\varepsilon_{P}^{eff}$ is the effective plastic strain.

#### 4.3.3. Rubber

Blatz–Ko rubber [24] incorporates elements from both the Blatz and Ko methods, as described by a hyper-elastic rubber model that utilizes the type II Piola–Kirchoff stress. This model is particularly effective in accurately representing the behavior of compressible rubber materials, and it can be expressed mathematically using the Blatz–Ko strain energy density function:

(15) $$W=\frac{1}{2}G\left(\frac{I_{2}}{I_{3}}+2\sqrt{I_{3}}-5\right).$$

In this function, various variables are used. The shear modulus at infinitesimal deformation is represented by *G*, while *E* represents the Young’s modulus of elasticity and *υ* denotes the Poisson’s ratio. The invariant of the Cauchy–Green deformation tensor, denoted by *I_n_*(where *n* is equal to 1, 2, or 3), is also incorporated into the equation. Notably, Equation (15) contains the sole material constant, *G*, as show in Table 2.

#### 4.3.4. Supporting Structure

In order to simplify the computational model and save computational resources, the supporting structure of the reinforced concrete slab is modelled as a rigid body. The material parameters are shown in Table 2.

**Table 2:** Input parameters for concrete, steel, and rubber material models.

Material	Parameter	Value	Comments
Concrete	RO (Density)	2400 kg/m^3^	Material test data
	FPC (Uniaxial compression strength)	45.6 MPa	
	NPLOT	1	According to [23,25,26]
	INCRE	0	
	IRATE (Rate effects options)	1	
	Elements erode	1.1	
	DAGG (Maximum aggregate size)	24 mm	
	UNITS (Units options)	4	
Steel	Density	7800 kg/m^3^	Material test data
	Young’s modulus	2.09 × 10^5^ MPa	
	Poisson’s ratio	0.3	
	Yield stress	435.3 or 450.1 MPa	
Rubber	Density	1.27 kg/m^3^	According to [24,27]
	Poisson’s ratio	0.463	
	Shear modulus	24 MPa	
Supporting structure	Density	7800 kg/m^3^	According to [27]
	Young’s modulus	2.09 × 10^5^ MPa	
	Poisson’s ratio	0.3	

### 4.4. Parameter Setting

Workbench is a multi-physics simulation platform launched by Ansys, while LS-DYNA is the world’s most famous general-purpose explicit dynamics analysis programmer, which is especially suitable for the simulation of non-linear dynamical impact problems such as high-speed collisions, explosions, and metal forming. In this paper, the two are combined for transient dynamics simulation, using the DesignModeler module in Workbench for 3D modelling and the Mechanical module for meshing the model, while calling the Workbench material library for material assignment. The load is applied to the surface of the reinforced concrete slab in the form of the initial velocity of the impact module, without considering the motion state of the impact module before it separates from the piston rod. The LS-DYNA solver is used for calculation and LS-PREPOST is applied for post-processing.

The computational model defines face-to-face contact between the reinforced concrete slab and the rubber and support using keyword *CONTACT_AUTOMATIC_SURFACE_TO_SURFACE, and bounded contact between the counterweight steel plate and the impact cushion using *CONTACT_TIED_SURFACE_TO_SURFACE. To avoid zero energy modes, the keyword *HOURGLASS is defined to suppress the magnitude of the hourglass energy.

### 4.5. Defining Outputs

The main objective of a blast simulator is to apply an impact load to a test specimen that effectively simulates the real explosion load. Therefore, the output of the numerical calculations should include the two parameters of pressure and impulse. Assuming that the loading area is subjected to a uniformly distributed load, which is common practice for similar devices [18,28,29], the contact force generated by the mutual collision between the impact module and the test specimen is divided by the loading area to derive the pressure exerted by the impact module on the test specimen, and the impulse is obtained by integrating the P–t curve. According to the previously mentioned explosion loading Equations (12) and (13), by substituting the peak and impulse values of the load, the parameters of the real explosion can be calculated, such as the amount of charge *W*, the explosion distance *H,* and the proportional explosion distance *Z*.

## 5. Study of Impact Load Characteristics

The one-factor control variable method was used to establish the numerical schemes for different impact velocities, impact masses, and thicknesses of impact cushion materials, and the grey correlation analysis method was used to discuss the sensitivity of the influencing factors of peak load pressures and impulses.

### 5.1. The Shape of the Impact Module

**Figure 7:**
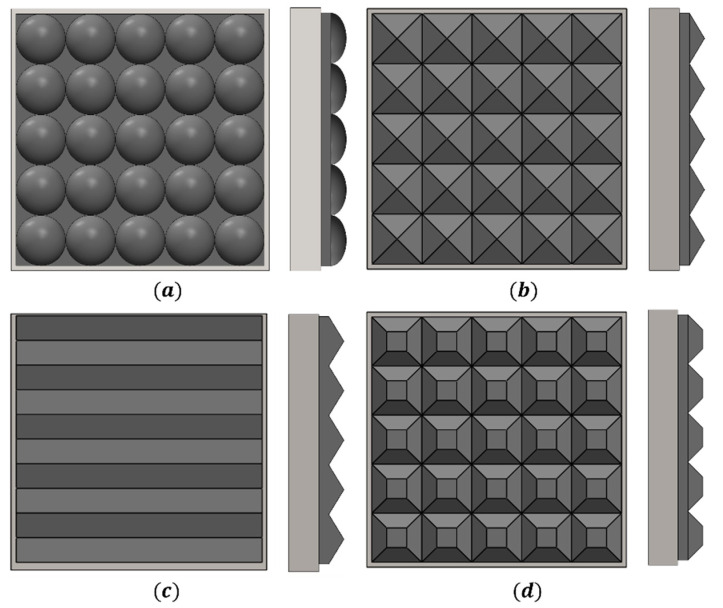
Schematic design of four impact cushion shapes: (**a**) Ellipsoidal; (**b**) Prismatic conical; (**c**) Prismatic; (**d**) Prismatic.

**Figure 8:**
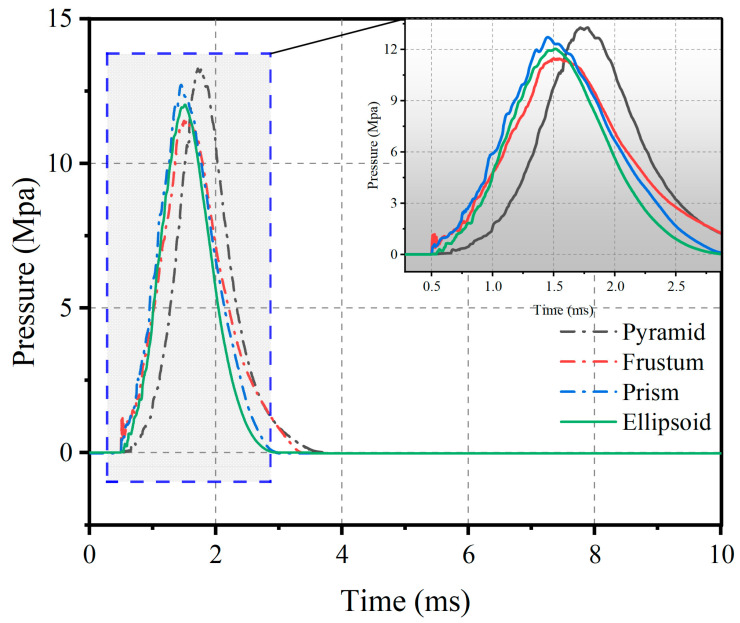
Pressure–time-course curves under four impact cushions.

The impact cushion plays a dominant role in regulating the impact load characteristics. In order to determine the optimum solution, four different shapes of impact cushion are designed as shown in Figure 7. The corresponding model was then constructed using numerical calculation, and the pressure–time curve was extracted at an impact speed of 20 m/s. The results are shown in Figure 8.

Comparing the impact loading curves under the four shapes of matting, the peak pressures and impulses are relatively similar. However, the prism shape produces significant oscillations, and the oscillations increase with the increase in the number of prisms [20]; the other three shapes of bedding have relatively smooth loading time courses under impact, but prismatic and prismatic rubber, in direct contact with reinforced concrete slabs, produce large stress concentrations. Therefore, the ellipsoidal shape was chosen for further analysis.

### 5.2. Effect of Flat Rubber Thickness

**Figure 9:**
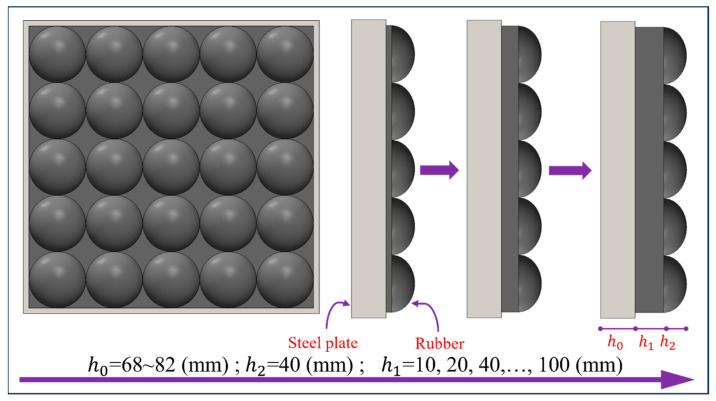
Schematic diagram of the variation of the flat rubber thickness.

In order to investigate the correlation between flat rubber thickness *h*_1_ and impact loading, the flat rubber thickness *h*_1_ in the impact module was adjusted and set in the range of 10 mm to 100 mm. At the same time, ellipsoid rubber thickness was kept at 40 mm, and the thickness of the counterweight steel plate was changed accordingly to ensure that the total mass of the impact module remained constant, as shown in Figure 9. According to this design scheme, six impact modules with different flat rubber thicknesses were designed and they were applied to the RC plate separately using the same velocity of 20 m/s to investigate the correlation between the flat rubber thickness and the impact loading.

**Figure 10:**
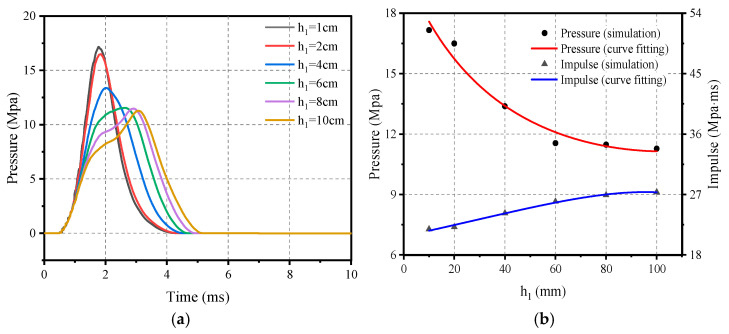
Influence of flat rubber thickness on the loading characteristics: (**a**) Pressure–time-course curve; (**b**) Peak pressure and impulse.

Figure 10 clearly shows the pressure–time-course curves and the corresponding peak load trends for the six flat rubber thicknesses. It can be clearly seen that the peak pressure gradually decreases and the impulse gradually increases as flat rubber thickness *h*_1_ increases. It is worth noting that this trend starts to diminish when the flat rubber thickness *h*_1_ reaches 60 mm. Overall, the relationship between the thickness of the screed rubber and the loading characteristics can be expressed as Equations (16) and (17).

(16) $$P_{h_{1}}=20.234/\left(1+0.158\times h_{1}-0.0076\times h_{1}{}^{2}\right),\;\;\;10\;\mathrm{mm}\leq \times h_{1}\leq 100\;\mathrm{mm}$$

(17) $$I_{h_{1}}=\left(20.776-0.959\times h_{1}\right)\left(1-0.085\times h_{1}+50141^{-7}\times h_{1}{}^{2}\right),\;10\;\mathrm{mm}\leq h_{1}\leq 100\;\mathrm{mm}$$

### 5.3. Effect of Ellipsoid Rubber Thickness

**Figure 11:**
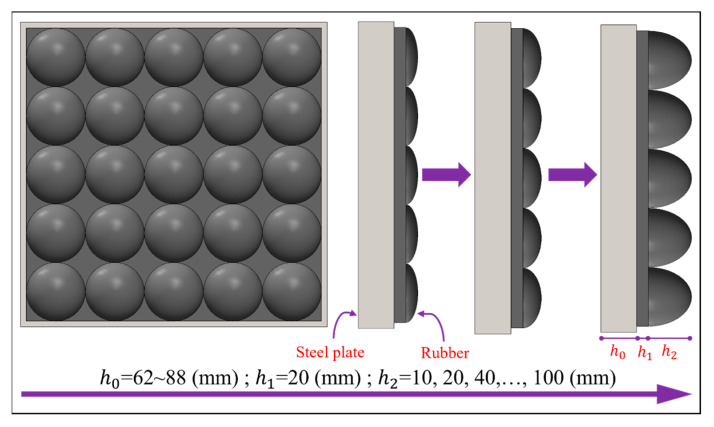
Schematic diagram of ellipsoid rubber thickness variation.

In order to further investigate the correlation between ellipsoid rubber thickness *h*_2_ and the impact loading, the ellipsoid rubber thickness *h*_2_ in the impact module was adjusted to a range of 10 mm to 100 mm, and flat rubber thickness *h*_1_ was kept at 20 mm, while the thickness of the counterweight steel plate was changed accordingly to ensure that the total mass of the impact module remained constant. Based on this principle, six different ellipsoid thicknesses of the impact module were designed, see Figure 11, and they were applied to the RC plate using the same velocity of 20 m/s to examine the correlation between the ellipsoid rubber thickness and the impact loading.

**Figure 12:**
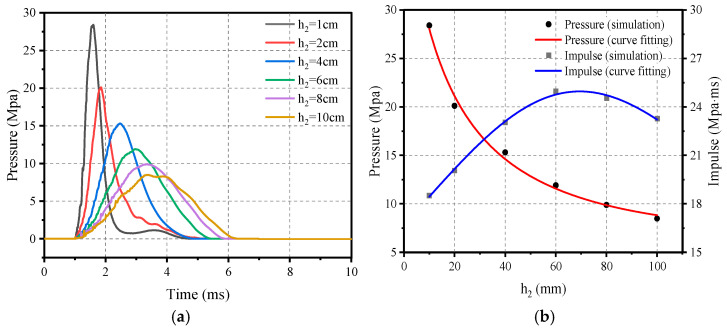
Effect of ellipsoid rubber thickness on loading characteristics: (**a**) Pressure–time-course curve; (**b**) Peak pressure and impulse.

As shown in Figure 12, it can be found that with the increase in ellipsoid thickness *h*_2_ the peak pressure gradually decreases, but the impact loading time is prolonged and, at the same time, the impulse also increases accordingly. It is worth noting that when the ellipsoid thickness *h*_2_ reaches 60 mm the peak pressure decreases, and the impulse begins to show a decreasing trend. This correlation can be illustrated by Equations (18) and (19).

(18) $$P_{h_{2}}=43.124/\left(1+0.0554h_{2}-1.65h_{2}{}^{2}\right),\;\;10\;\mathrm{mm}\leq h_{2}\leq 100\;\mathrm{mm}$$

(19) $$I_{h_{2}}=\left(16.687+0.047h_{2}\right)\left(1-0.008h_{2}+6.874^{-6}h_{2}{}^{2}\right),\;\;10\;\mathrm{mm}\leq h_{2}\leq 100\;\mathrm{mm}$$

### 5.4. Effect of Impact Velocity

In order to investigate the effect of velocity on the impact load characteristics, an impact module with a mass of 200 kg was selected and the velocity range was set between 10 m/s and 40 m/s for numerical calculations. In this process, the flat rubber thickness of the impact cushion is set to 20 mm and the thickness of the ellipsoid rubber is set to 40 mm.

**Figure 13:**
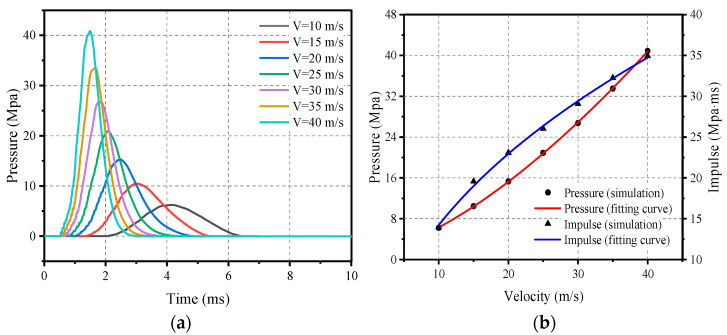
Effect of impact velocity on load characteristics: (**a**) Pressure–time-course curve; (**b**) Peak pressure and impulse.

Figure 13 demonstrates the load profile and the relationship between peak pressure and impulse, respectively, and velocity. It can be clearly observed that the peak pressure gradually increases with increasing velocity and the load time decreases accordingly; this correlation can be modelled and described by Equation (20). In addition, impulse also increases with velocity, although unlike pressure the rate of increase in impulse tends to decrease. This relationship can be accurately described by Equation (21).

(20) $$P_{v}=1.478+0.148\cdot V^{1.513},\;\;\;10\;m/s\leq V\leq 40\;m/s$$

(21) $$I_{v}=-10.821+9.264\cdot V^{0.432},\;\;\;\;10\;m/s\leq V\leq 40\;m/s$$

### 5.5. Effect of Impact Module Quality

**Figure 14:**
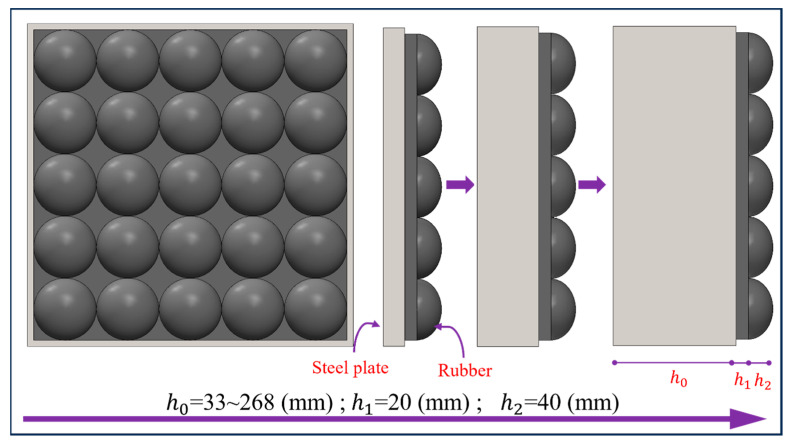
Schematic diagram of thickness variation of counterweight steel plate.

Similarly, the thickness of the flat rubber is 20 mm and the thickness of the ellipsoid is 40 mm in the elastic cushion, while the thickness of the counterweight steel plate is changed to design the impact module with different masses ranging from 100 kg to 600 kg as shown in Figure 14. The impact modules with different masses were applied to the RC plate using the same velocity of 20 m/s to investigate the relationship between mass and impact load.

**Figure 15:**
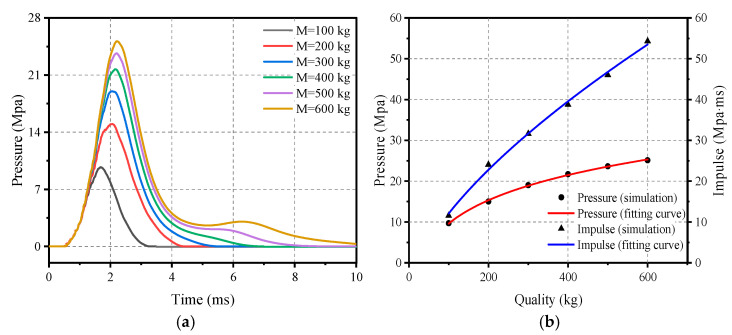
Effect of impact mass on load characteristics: (**a**) Pressure–time-course curve; (**b**) Peak pressure and impulse.

Figure 15 depicts a visual representation of the effect of the mass of the impact module on the loading characteristics. It can be clearly seen that as the mass increases both the peak pressure and the impulse begin to grow; this trend can be represented by Equations (22) and (23).

(22) $$P_{M}=-64.107+44.875M^{0.108},\;\;\;100\;\mathrm{kg}\leq M\leq 600\;\mathrm{kg}$$

(23) $$I_{M}=-6.747+0.951M^{0.649},\;\;\;100\;\mathrm{kg}\leq M\leq 600\;\mathrm{kg}$$

## 6. Sensitivity Analyses of Influencing Factors

### 6.1. Relevance Calculation

Based on the results of the above numerical calculations, the change value of each influence parameter is selected as the comparison data matrix X, and the load P under the corresponding conditions is used as the reference data matrix; the comparison data matrix and the reference data matrix are established, respectively, to calculate the grey correlation of the factors influencing the peak pressure of the impact load.

(24) $$X=\left(\begin{array}{l} X_{1}\\ X_{2}\\ X_{3}\\ X_{4} \end{array}\right)=\left(\begin{array}{cccccc} 10 & 15 & 20 & 25 & 30 & 35\\ 100 & 200 & 300 & 400 & 500 & 600\\ 10 & 20 & 40 & 60 & 80 & 100\\ 10 & 20 & 40 & 60 & 80 & 100 \end{array}\right)$$

(25) $$Y=\left(\begin{array}{c} Y_{1}\\ Y_{2}\\ Y_{3}\\ Y_{4} \end{array}\right)=\left(\begin{array}{cccccc} 6.227 & 10.459 & 15.309 & 20.899 & 26.748 & 35.505\\ 9.713 & 15.004 & 19.013 & 21.714 & 23.661 & 25.135\\ 17.161 & 16.495 & 13.383 & 11.519 & 11.475 & 11.277\\ 28.411 & 20.113 & 15.308 & 11.903 & 9.892 & 8.481 \end{array}\right)$$

Pass the *X* and *Y* matrices through Equations (5) and (6). to obtain the difference sequence matrix ∆:

(26) $$\Delta =\left(\begin{array}{cccccc} 0 & 0.045 & 0.067 & 0.062 & 0.048 & 0\\ 0 & 0.143 & 0.203 & 0.178 & 0.104 & 0\\ 1 & 0.776 & 0.025 & 0.509 & 0.744 & 1\\ 1 & 0.472 & 0.009 & 0.384 & 0.707 & 1 \end{array}\right).$$

From Equations (7) and (8) the correlation coefficient matrix *L* can be obtained as

(27) $$L=\left(\begin{array}{cccccc} 1 & 0.428 & 0.333 & 0.351 & 0.413 & 1\\ 1 & 0.415 & 0.333 & 0.363 & 0.493 & 1\\ 0.35 & 0.411 & 1 & 0.52 & 0.422 & 0.35\\ 0.339 & 0.524 & 1 & 0.576 & 0.422 & 0.339 \end{array}\right).$$

Finally, from Equation (9) the correlation matrix *G_P_* is obtained as

(28) $$G_{P}={\left(\begin{array}{cccc} 0.587 & 0.601 & 0.509 & 0.533 \end{array}\right)}^{T}.$$

Similarly, the grey correlation of the factors influencing the impact load impulse can be calculated based on the numerical simulation results, and the correlation matrix *G_I_* is

(29) $$G_{I}={\left(\begin{array}{cccc} 0.683 & 0.696 & 0.646 & 0.565 \end{array}\right)}^{T}.$$

### 6.2. Sensitivity Assessment

Peak load pressure, in descending order of sensitivity, is flat rubber thickness *h*_1_, ellipsoid rubber thickness *h*_2_, impact velocity *V,* and impact modulus mass *M*. Unlike pressure, impulse is more sensitive to changes in flat rubber thickness *h*_1_ than in ellipsoid rubber thickness *h*_2_; load impulse, in descending order of sensitivity, is impact modulus mass m and impact velocity *v*. The sensitivities are impact modulus mass *m* and impact velocity *v*. The sensitivities are impact modulus mass m and impact velocity *v*, flat rubber thickness *h*_1_ and ellipsoid rubber thickness *h*_2_.

## 7. Summary and Conclusions

In this study, the experimentally calibrated finite element model was rationalized and numerically calculated for four variables, while grey correlation analysis was applied to further assess the sensitivity of the impact loads to different parameters. Within the scope of this study, the main parameters investigated include the four parameters of impact velocity, impact module mass, flat rubber thickness, and ellipsoid rubber thickness. The following conclusions can be drawn from the numerical studies presented in this paper:

(1) Changes in ellipsoid rubber thickness had a more positive effect on impact loading than flat layer rubber thickness. It is worth noting that, when the ellipsoid thickness increased from 10 mm to 100 mm, the peak pressure showed a maximum decrease of 29%, and the average decrease was maintained at 21%. The impulse also increased from the initial growth, and showed a decreasing trend at the later stage; however, during the growth of the flat layer rubber thickness from 10 mm to 100 mm, the peak pressure showed a maximum decrease of 19% and the average decrease was only 8%, and the impulse increased gradually and the average increase was 5%.
(2) The impact velocity has a significantly greater effect on the peak pressure of the impact load than the impulse. When the velocity increases from 10 m/s to 15 m/s the peak pressure increases by 68% and the impulse increases by about 40%. However, after that, when the speed increases every 5 m/s the peak pressure can still keep a high increase of 46%. It maintains this increase until the speed reaches 40 m/s, where it can still maintain a minimum increase of 22%. The increase in impulse is rapidly reduced to 18%. When the speed increases to 40 m/s, the impulse only increased by 8%.
(3) Unlike impact velocity, the mass of the impact module had a significantly greater effect on the impact load impulse than the peak pressure. When the mass increased from 100 kg to 200 kg the peak pressure increased by 54% and the impulse increased by about 108%. However, thereafter, as the mass increases by 100 kg, the increase in peak pressure decreases to 27%. It continues to decrease until the mass increases to 600 kg, where the increase is only 6%. Meanwhile, the increase in impulse decreases rapidly but still retains an increase of 32%. When the mass increases to 600 kg, the impulse also maintains a minimum increase of 18%.
(4) When the four factors of impact module mass, impact velocity, ellipsoid rubber thickness, and flat rubber thickness are changed the peak load pressure and impulse can be affected. Peak load pressure and impulse are sensitive to changes in these factors, but there are differences in the degree of sensitivity to the thickness of the ellipsoid rubber and flat rubber thickness. Specifically, both peak load pressure and impulse are most sensitive to the mass of the impact module. The difference is that peak pressure is more sensitive to changes in ellipsoid rubber thickness than flat rubber thickness, while the opposite is true for impulse.

## Figures and Tables

**Table 1:** The process of impact.

T = 0	$0\;\text{<}\;T\;\text{<}\;T_{i}^{-}$	$T=\;T_{i}^{-}$	$T=T_{i}$
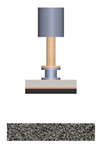	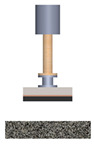	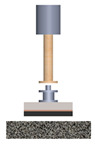	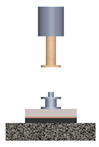
Initial	Acceleration	Separation	Impact

## Data Availability

The data presented in this study are available on request from the corresponding author.
